# Insights into *Mycobacterium leprae* Proteomics and Biomarkers—An Overview

**DOI:** 10.3390/proteomes9010007

**Published:** 2021-01-29

**Authors:** Sakshi Gautam, Devesh Sharma, Anjana Goel, Shripad A. Patil, Deepa Bisht

**Affiliations:** 1 Department of Biochemistry, National JALMA Institute for Leprosy and Other Mycobacterial Diseases, Tajganj, Agra 282004, India; sakshigautam626@gmail.com (S.G.); devbiotech89@gmail.com (D.S.); shripadpatil@yahoo.com (S.A.P.); 2 Department of Biotechnology, GLA University, NH-2, Mathura-Delhi Road, Mathura 281406, India; anjana.goel@gla.ac.in

**Keywords:** biomarkers, diagnosis, leprosy, proteomics, vaccine

## Abstract

Although leprosy is curable, the identification of biomarkers for the early diagnosis of leprosy would play a pivotal role in reducing transmission and the overall prevalence of the disease. Leprosy-specific biomarkers for diagnosis, particularly for the paucibacillary disease, are not well defined. Therefore, the identification of new biomarkers for leprosy is one of the prime themes of leprosy research. Studying *Mycobacterium leprae*, the causative agent of leprosy, at the proteomic level may facilitate the identification, quantification, and characterization of proteins that could be potential diagnostics or targets for drugs and can help in better understanding the pathogenesis. This review aims to shed light on the knowledge gained to understand leprosy or its pathogen employing proteomics and its role in diagnosis.

## 1. Introduction

Despite advances toward the elimination of leprosy over the last four decades, leprosy still remains an important health problem [1,2]. It is a treatable infection that ranks as the second most pathogenic mycobacterial infectious disease after tuberculosis. Leprosy is the clinical manifestation of a dermatoneurological disease caused by the yet-uncultured pathogen *Mycobacterium leprae*. Despite effective multidrug therapy (MDT), the torpid decline in new leprosy cases demonstrates that transmission in the society is persistent. In 2018, new diagnosed cases were 208,619, and India alone accounted for more than half of new cases reported globally [3]. Recently, Kundakci and Erdem [4] described leprosy as a great imitator joining syphilis, mycosis fungoides, etc. Moreover, mathematical modeling predicts that millions of linger undetected [5]. Persistent incidence is commonly assigned due to undetected or undiagnosed subclinical cases.

*M. leprae* possesses a longer generation time and lacks an artificial medium for in vitro growth; therefore, animals are used for in vivo propagation of bacilli [6]. Nine-banded Armadillo is widely accepted animal model, and besides this, other animals (rat, mice, guinea pig, etc.) used in the laboratory are immunologically resistant, and hence confined growth appear in specific regions such as the footpad [7,8]. Later in 2016, in the British Isles region, leprosy-like lesions were noticed in red squirrels (*Sciurus vulgaris*), and their existence was confirmed by the *M. leprae* and *M. lepromatosis* genome in the animal. Two modes of transmission of leprosy viz. anthroponotic and zoonotic were discussed. The transmission of *M. leprae* may occur from the reservoir (epidemiologically connected populations or environment) to the target population. Transmission from an animal reservoir to the environment involves interconnection through an ecological cycle. Transmission and reservoir of the *M. leprae* complex might assist in understanding the pathogenesis of the disease [9,10]. The World Health Organization(WHO )as delineated objectives to stop the transmission of new leprosy cases between 2016 and 2020, and the development of new diagnostic tools is emphasized to be of utmost importance [11].

## 2. *Mycobacterium leprae*

*Mycobacterium leprae* is a rod-shaped, acid-fast, non-motile, non-spore forming, slow-growing (generation time 12–14 days), obligate intracellular pathogen that affects mainly peripheral nerves and skin, leading to nerve damage and disfigurement. It might also affect other body parts such as bone marrow, liver, spleen, lymph nodes, lungs, oesophagus, kidney, eyes, and testes in human leprosy [12,13]. It cannot be cultivated under in vitro conditions; however, it can be propagated in nine-banded armadillo (*Dasypus novemcinctus*) or footpads of the mouse or cooler parts of host, especially human [14]. *M. leprae* has the smallest genome (3.3 Mb) among mycobacteria with 1614 protein-encoding genes and remarkable 1300 pseudogenes [15,16,17]. As a result of reductive evolution, which is the hallmark of *M. leprae*, it has become host-associated [18]. Despite massive gene decay, *M. leprae* has managed a minimal gene set that allows its survival within the host. Since the availability of the *M. leprae* genome sequence, various studies have focused on identifying genes encoding *M. leprae*-unique antigens to design new diagnostic tests [19].

## 3. Vaccine

To date, Bacillus Calmette Guerin (BCG) is the only vaccine being used against mycobacterial diseases tuberculosis and leprosy [20]. *Mycobacterium indicus pranii* (MIP), an indigenous vaccine developed by the Indian National Institute of Immunology, New Delhi is another vaccine that has shown promising results in hospital and population-based trials against leprosy. It reduces the bacillary load; completes clearance of granuloma; reduces reactions, neuritis, and MDT duration; and it upgraded lesions histopathologically in leprosy patients [21,22,23]. Presently, a field project is being undertaken by Indian Council of Medical Research ICMR) and National Leprosy Eradication Programme (NLEP) in which the MIP vaccine is given to the index leprosy patient beside MDT. The family members and contacts are also being immunized with vaccine twice at a six-month interval. The vaccine is believed to boost the immune system against the leprosy disease.

Another vaccine candidate for leprosy is LepVax (LEP-F1 + GLA-SE), whose phase I antigen dose-escalation trial related to safety, tolerability, and immunogenicity has been recently conducted in healthy adults. It is safe and immunogenic in healthy individuals, and the authors supported its testing in leprosy endemic regions [24]. LepVax is a cocktail of recombinant polyprotein LEP-F1 (ML2055, ML2380, and ML2028) with GLA-SE (Glucopyranosyl Lipid Adjuvant in the stable emulsion) adjuvant formulation. Duthie et al. suggested that post-exposure prophylaxis with LepVax is not only safe but also alleviates and delays the neurological disturbances triggered by *M. leprae* infection unlike BCG [25].

## 4. Diagnosis

Diagnosis before clinical manifestations is vital to the reduction of transmission. Recent strategies to stop leprosy transmission rely on prophylactic protocols using rifampicin and/or BCG [26]. The diagnosis of leprosy is currently dependent on the clinical signs and symptoms, which include anesthetic skin lesion(s), enlarged peripheral nerve(s), and the presence of acid-fast bacilli in the skin smear, and histopathology is performed for the confirmation of a clinically doubtful case. So far, different types of classifications were proposed for leprosy patients; nevertheless, two foremost classifications are the Ridley Jopling [27] and the WHO proposed classification [28]. The Ridley Jopling classification was based on the bacteriological index (BI), clinical, histopathological, and immunological features. Here, two forms were polar stable, while the borderline in between the two was unstable. The spectrum starts with Tuberculoid leprosy (TT), Borderline tuberculoid leprosy (BT), Mid-borderline leprosy (BB), Borderline lepromatous leprosy (BL), and Lepromatous leprosy (LL); however, the WHO classification was based on BI (bacillary load in the patient slit skin smear) or the number of skin lesions (in the absence of slit skin smear). The two categories were paucibacillary tuberculoid leprosy (PB, number of skin lesions are 1 to 5 and skin smear-negative) and multibacillary lepromatous leprosy (MB, number of skin lesions are >5 and skin smear positive) [29,30]. The National Leprosy Eradication Programme (NLEP) 2009 of the Indian government considered the nerve involvement criteria for classification. For PB patients, these criteria were no nerve or only one peripheral nerve involved with or without one to five lesions and negative skin smear, while for MB patients, the criteria were more than one peripheral nerve involved regardless of the number of skin lesions and a negative skin smear [31].

Several attempts have been made for the development of specific tests for the early detection of leprosy but with little success. Various assays that detect leprosy-specific antibody responses such as ELISAs [32], the *M. leprae* gelatin particle agglutination test [33], the dipstick test [34], and the lateral flow test [35] have been developed. Serological test detecting IgM antibodies against phenolic glycolipid-1 (PGL-1) is useful in multibacillary but not in identifying paucibacillary patients. The Mitsuda skin test is also not specific, as it can be mediated by lymphocytes responsive to *M. tuberculosis*. The limitation of the use of interferon-γ (IFN-γ) for diagnosis is that individuals with adequate immunity against *M. leprae* also produce substantial concentrations of IFN-γ. Palit and Kar have nicely reviewed the current scenario on the prevention of transmission of leprosy [36]. The Netherlands Leprosy Relief has proposed an enhanced PEP++ regimen. Recently, Leturiondo et al. [37] evaluated the performance of PGL-1 and natural disaccharide octyl—Leprosy Infectious Disease Research Institute Diagnostic-1 (NDO-LID) in the discrimination of leprosy cases from healthy individuals. However, the test showed limited capacity in diagnosis. Molecular techniques such as PCR, *M. leprae*-specific repetitive element (RLEP), and real-time PCR have been used to detect the components of *M. leprae* in the patient lesions or household contacts. None of the tests was successful in detecting early leprosy. One of the major obstacles in the early diagnosis of leprosy is the lack of good markers. Proteomics is a very powerful technology for biomarker discovery in many diseases [38], and during the past couple of years, proteomic research has grown remarkably and seen unprecedented development due to technological advancement. Recently, Sengupta [39] reviewed the recent advances in *M. leprae*-specific tests for the early diagnosis of leprosy.

## 5. Proteomics

Proteomics is the global analysis of proteins expressed in a cell or tissue or an organism. It is more complicated compared to genomics, as an organism’s genome is more or less constant, whereas the total protein expression profile changes with time and is also influenced by environmental conditions. Nucleic acid-based systems offer rapid and sensitive methods to detect the presence of genes; however, developments in molecular and cellular biology have imposed doubts on the ability of genetic analysis alone to predict any complex phenotypes [40,41]. In addition, one gene can code for several proteins. Thus, it not only provides the opportunity to determine the functional genome but also facilitates the identification of proteins that have not been predicted by genome analysis.

Proteomics has been extensively used for both basic as well as translational research in the areas of infectious diseases, diabetes, cancers, cardiovascular disease, etc. Proteomics can either be qualitative (analytical) or quantitative. The major steps involved in analytical proteomics are isolation, separation following digestion into peptides or vice versa, and identification. After the isolation of proteins, separation is usually done by two-dimensional gel electrophoresis (2DGE) or various chromatography-based approaches. Despite landmark progress made in the development of alternative protein separation techniques, 2DGE is still a powerful technique to study proteins. Peptides generated as a result of enzymatic digestion are analyzed by mass spectrometry (MS), either MALDI-TOF or ESI, and data generated thereafter are matched with available databases using various bioinformatics software. During the past couple of years, much advancement has been made in the field of proteomics. The development of sensitive, rapid, and powerful MS-based methods have resulted in the accurate identification, quantification, and modification of any expressed protein. Quantitative proteomics could be useful both for the early detection of diseases and evaluation of pathological status [42,43]. Non-gel-based proteomic experiments are an ideal platform for the identification of proteins. Proteins that exhibit an increase or decrease in abundance between distinct proteomes are potential biomarkers. Different techniques have been developed to simultaneously compare protein levels across multiple samples. One method that has gained increased attention is iTRAQ, which is a shotgun technique that uses Isobaric Tags for Relative and Absolute Quantitation. Compared to other methods such as 2DGE, ICAT (isotope-coded affinity tags), and DIGE (differential gel electrophoresis), iTRAQ offers improved quantitative reproducibility and higher sensitivity [44]. Various tools and techniques both classical and newly emerging to study proteomics primarily 2DGE, MS and protein microarrays have been reviewed [45,46]. Recently, our group has also reviewed the development and advancement in technology in the field of proteomics and the pivotal role it played in answering many unexplored questions related particularly about tuberculosis [47].

## 6. Proteomics and *Mycobacterium leprae*

Leprosy is one of the infectious diseases that has also been benefitted by proteomics. Several developments have been made toward the identification of *M. leprae* proteins employing proteomics tools. Knowledge gained on the biology and pathogenesis of *M. leprae* from proteomic studies has been reviewed by Prakash and Singh [48]. The employment of modern proteomics tools toward the proteomics of leprosy bacillus was reported by Pessolani et al. [49]. On analyzing the cell extract by one-dimensional gel electrophoresis, three new proteins were detected. However, analyzing the cytosolic fraction by 2DGE resulted in a greater number of proteins. Marques et al. [50] carried out the proteomic analysis of armadillo-derived *M. leprae* subcellular fractions employing 2DGE and mass spectrometry. This was the first study where the application of proteomics has been extended to a host-derived Mycobacterium. In total, 147 protein spots corresponding to 44 genes were identified, and 28 were found to be new proteins. Furthermore, two highly basic proteins with pI more than 10 were isolated, employing heparin affinity chromatography. For some time, in silico tools were mainly used for the identification of antigens, and proteomic approaches have not been explored to study *M. leprae*. Wiker et al. [51] were the first to re-analyze their previous data and answer many questions related to antigen prediction and pseudogene expression. They argued that combining proteomic approaches with bioinformatics workflows is a required step in the characterization of important pathogens.

Marques et al. [52] discussed the potential role of *M. leprae* proteins as biomarkers and resolved 391 proteins employing 2DGE from three cellular fractions viz. the cell wall, membrane, and cytosol. A total of 14 protein spots were identified, and among these, eight protein spots were identified based on reactivity with monoclonal antibodies and relative size/pI, while six protein spots were identified by microsequencing. They eventually identified new proteins—elongation factor EF-Tu and *Mycobacterium tuberculosis* (*M. tb*) MtrA response regulator. In another study, they [53] deciphered the proteome of the *M. leprae* cell envelope employing a high-throughput proteomic approach and identified 218 new *M. leprae* proteins. The proteins were mainly enzymes involved for lipid biosynthesis and degradation, the biosynthesis of major components of the mycobacterial cell envelope, proteins involved in transportation across lipid membranes, and lipoproteins and transmembrane proteins with unknown functions. The identification of proteins expressed in vivo by the bacillus will be of great significance in understanding the mycobacterial pathogenesis. Silva et al. [54] studied the nude mouse-derived *M. leprae* cell surface-exposed proteome to unravel potentially relevant adhesins and highlighted the role of adhesins in bacillus–epithelial cell interaction. A total of 279 cell surface-exposed proteins were identified by shotgun mass spectrometry. Rana and co-workers [55] presented a proteome-wide identification of surface-associated and secretory proteins (SASPs), which are considered as attractive targets against bacterial pathogens and identified 17 lipoproteins, 11 secretory, and 19 novels OMPs (outer membrane proteins) in *M. leprae*. They suggested that 11 OMPs with B-cell epitopes may be considered as important candidates for developing immunotherapeutics against *M. leprae*.

## 7. Biomarkers in Leprosy

Biological fluids from patients and controls are a reliable source for the identification of protein markers. Serum/plasma proteome is complex but offers an important window on individual variation. Serological biomarkers of infection, disease progression, and treatment efficacy for leprosy have been studied. Patil and co-workers [56] studied serum proteins in leprosy by polyacrylamide gel electrophoresis. Gupta et al. [57] analyzed the two-dimensional proteome profiles of serum from leprosy patients and showed differential expression of the acute-phase protein haptoglobin isoform specifically in erythema nodosum leprosum (ENL) patients. They further reported the differential expression and glycosylation of another acute-phase protein, α1-acid glycoprotein (AGP), in ENL cases by 2DE and ELISA [58] compared with healthy controls and speculated on the possible role of AGP in the ENL stage of leprosy. Mendes et al. [59] reported that pentraxin 3 (PTX3) is enhanced during ENL but not in reversal reaction and suggested a new molecular target in ENL pathogenesis. The TlyA protein of *M. leprae* was found to be a probable biomarker of active infection [60]. Soares et al. [61] suggested the potential of aldo-keto reductase family 1 member B10 (AKR1B10) as a biomarker and therapeutic target in Type 2 reaction. Recently, Barbosa et al. [62] indicated the utility of recombinant protein rMLP15 in the diagnosis of leprosy. Manta et al. [63] reported Quantitative PCR for leprosy diagnosis and monitoring in household contacts. Spencer et al. [64] used antibody titers against specific *M. leprae* antigens such as PGL-1, lipoarabinomannan, and four recombinant protein in understating the dynamics of patient antibody responses during and after drug therapy. This could assist in monitoring the treatment efficacy in leprosy patients and assess the disease progression of those who are at risk of developing the disease.

As multiple factors such as bacterial, genetic, environmental, and nutritional contribute to clinical manifestations, studies related to metabolites from the serum of persons affected with leprosy were carried out [65]. Three polyunsaturated fatty acids (PUFs) involved in the inhibition of inflammation were present in higher levels in cases of lepromatous leprosy. Silva and Belisle [66] discussed the possible consequences and new hypotheses for the involvement of ω3 and ω6 PUFA metabolism in the pathogenesis of leprosy. ω6 PUFA, arachidonic acid (AA) is the precursor for a variety of lipid mediators such as prostaglandins, thromboxanes, leukotrienes, lipoxins, etc. that exhibit immune-inflammatory functions. Vardhini and co-workers [67] utilized bioinformatic tools to understand leprosy nerve damage by performing sequence and structural similarity searches of myelin P0, a major peripheral nerve protein, with leproma and another genomic database. They inferred that it could be important in molecular mimicry, receptor binding, and cell signalling events involved in neurodegeneration. A rise in the levels of autoantibodies and T cell response to cytoskeletal proteins in leprosy was demonstrated by Singh et al. [68] employing 2DGE, Western blot, and MALDI-TOF/TOF mass spectrometry. This group further reported a tropomyosin-mimicking ATP-dependent Clp protease ATP-binding subunit of *M. leprae* that might be responsible for extensive tissue damage during type 1 reaction. Owing to their small size, peptides can be expressed on the surface of bacteriophage to select mimicking peptides from different targets. Alban et al. [69] suggested that mimetic peptides might have important applications in the diagnosis of leprosy because of their versatility to perform the same functions as the natural antigens.

Urinary signatures as biomarkers in case of leprosy were first reported by Mayboroda et al. [70]. The group suggested that urinary metabolome could be used to discriminate between controls and patients. Moreover, metabolic signatures of patients developing reversal reaction (RR) were different before the onset of RR compared to at RR diagnosis. Using multiplex-bead-arrays, [71] identified new biomarkers (ApoA1, IL-1Ra, S100A12) for leprosy, confirmed five previously described biomarkers (CCL4, CRP, IL-10, IP-10, αPGL-1 IgM), and assessed their applicability in point-of-care (POC) tests. Blood coagulation abnormalities were reported in multibacillary leprosy patients by da Silva [72]. Differential 2D-proteomics analysis between leprosum and control clots exhibited two proteins, complement component 3 and 4 and inter-alpha-trypsin inhibitor family heavy chain-related protein (IHRP), in only leprosy patients’ clots. The group argues that some components such as fibrinogen might be potential predictive biomarkers of leprosy reactions.

Stefani et al. [73] describe the cellular immune response to type Th1/IFN-γ and serology tests that could be used to diagnose patients with PB and MB. The patient with PB has a high cellular immune response (RIC) type Th1/IFN-γ and a low level of antibodies, whereas the patient with MB has a vice versa reaction. This study reveals the early diagnosis of PB and MB cases. In addition to new recombinant proteins, the PGL-I antigen has been found to enhance the serological diagnosis of PB and MB patients. New fusion proteins, including the most immunogenic antigens of *M. leprae*, such as the Leprosy Infectious Disease Research Institute Diagnostic-1 (LID-1) antigen, provide the possibility of producing chimeric antigens that could provide greater sensitivity for the identification of MB and possibly PB patients. Few studies are underway to determine the immunoreactivity and specificity of new antigens that can be integrated into the PGL-1 antigen, intending to obtain a higher seropositivity test between MB and PB patients [74,75,76]. Recently, Santos et al. [77] reported that IL-17A and IL-1β concentration is higher in PB than MB patients serum. However, in MB patients, higher serum concentrations of IFN-γ than PB developed leprosy reactions (MB LR). Th17 cells were associated with an efficient inflammatory response that was present in the PB type but was not predictive of leprosy reactions in MB patients. A list of identified biomarkers for the diagnosis of *M. leprae* infection in various groups is provided in Table 1.

Benjak et al. [78] studied the phylogenomics and antimicrobial resistance of the leprosy bacillus. DNA was extracted using a customized protocol from the patients’ skin biopsies. In addition to the known MDT-resistance mutations, the group was able to detect other mutations linked with antibiotic resistance. Recently, de Macedo et al. [79] reviewed the implications of metabolic changes on the course of *M. leprae* infection, which might play a significant role in immune modulation in leprosy. Contacts of leprosy patients are a population at high risk of contracting and suffering from the effects of the disease during their lifetime. They can also act as *M. leprae* carriers and therefore serve as sources for transmission and infection. Being important links in the chain of transmission, several epidemiological studies [80,81] with household contacts have been conducted. Romero-Montoya et al. [82] suggested that a follow-up of household contacts is a good strategy for the early diagnosis of leprosy and to monitor transmission. The development of highly and sensitive diagnostic methods to screen this population is currently needed.

### Origin and Functions of Some Biomarkers at a Glance

(a) Phenolic glycolipid-1 (PGL-1): It is specific to *M. leprae* and present mainly in the cell wall and capsule of the bacteria. It is highly specific due to the trisaccharide units and gets entered inside the cell by binding specifically to the G domain of the laminin a2 chain in the basal lamina of Schwann cell-axon units [83].
(b) Natural disaccharide octyl bovine serum albumin (ND-O-BSA) or human serum albumin (ND-O-HSA): It is the modified (conjugated with protein BSA), semisynthetic antigen representing the PGL-1 molecule of *M. leprae* developed later and is still in use. This antigen is superior to other derivatives of the PGL-I antigen [84,85]. An increased level of serum IgM antibodies against ND-O-HSA has been observed in MB patients [86].
(c) L-ESAT-6: Early secreted antigenic target-6 (L-ESAT-6): *M. leprae* ESAT-6 (L-ESAT-6) is the homologue of *M. tb* ESAT-6 (T-ESAT-6) having 36% similarity at an amino acid level. It is an important *M. leprae* antigen that stimulates T-cell dependent IFN-γ production in *M. leprae*-exposed individuals. Remarkable cross-reactivity was observed between T-ESAT-6 and L-ESAT-6, which suggests that L-ESAT-6 may play a crucial role in the diagnosis of leprosy [87,88]
(d) Leprosy IDRI diagnostic (LID-1): This marker was developed by the fusion of two selected proteins ML0405 and ML2331 (involved in the diagnosis of MB patients) and has been named LID-1 (Leprosy Infectious Disease Research Institute Diagnostic-1) [75]. A significant increment in the level of serum IgG1 and IgG3 antibodies against LID-1 was notified in MB patients [86].
(e) Natural disaccharide octyl and LID-1 (NDO–LID): As the name suggests, it is the conjugate of NDO and LID-1 into the single fusion complex. This complex possesses antibody-detecting capabilities of the individual antigens and is good for antibody-based detection for leprosy patients than singly [89]. An increment in the level of serum IgG1 and IgG3 antibodies against NDO–LID in MB patients was observed [86].
(f) Monocyte chemoattractant protein-1 (MCP-1) or CCL2: It is a signaling molecule secreted by monocytes, memory T cells, and recruiting other immune cells to the sites of inflammation and infection. An increased level of this chemokine has been observed in leprosy patients than in healthy individuals [90].
(g) Macrophage inflammatory protein-1β (MIP-1β) or CCL4: It acts as a chemo-attractant biomarker for monocytes, and it inhibits T cell activation through TCR signaling [91]. The function of MIP-1β in leprosy pathogenesis is still unclear [92].
(h) Platelet-derived growth factor-BB (PDGF-BB): These molecules are processed by SSV-transformed or PDGF-B expressing cells. There are two genes viz. PDGF-A and PDGF-B which encode three proteins—PDGF-AA, PDGF-AB, and PDGF-BB—comprising PDGF family [93]. PDGF-BB represents one of the promising markers of T2R [94].
(i) Interleukin-1β (IL-1β): It is a pro-inflammatory cytokine that is linked with inflammasome development and is crucial for Th17 cells differentiation [95]. Liu et al. [96] reported a significantly decreased expression level of the IL-1β gene in LL patients.

## 8. Performance of Biomarkers

Potential biomarkers aid in understanding the mechanisms of leprosy reactions and diagnosed the clinical stages. The elevated level of circulating cytokines CXCL10 and IL6 act as promising markers for leprosy in T1R. Similarly, IL7 and PDGF-BB represent potential markers of T2R [94]. Medeiros et al. [111] describe that CXCL10, CCL2, and matrix metalloproteinase 2 and 9 (MMP2 and MMP9) immunoreactivities were found in the leprosy nerves but not in non-leprosy samples. *M. leprae*-unique Ags, particularly ML2478, act as biomarker tools to measure *M. leprae* exposure using IFN-γ or IFN-inducible protein-10, and they also show that MCP-1, MIP-1β, and IL-1β can potentially distinguish pathogenic immune responses from those induced during asymptomatic exposure to *M. leprae* [92]. Reduced expression of the IL-1β gene has been identified in patients with LL lesions. Patients with TT/BT generate more IL-1β in response to *M. leprae* [96]. MIP-1β (or CCL4) is a potential immunological biomarker that can inhibit T cell activation by interfering with TCR signaling [91]. An immunodominant antigen PGL-1 can produce a strong immune humoral response. The percentage of seropositivity was much higher in newly untreated multibacillary (MB) patients (83.9%) than in paucibacillary (PB) patients (17.8%) [112]. Geluk et al. [87] studied that *M. leprae* antigen L-ESAT-6 (Early secretory antigenic target 6) stimulates T-cell-dependent gamma interferon production in a large proportion of individuals exposed *to M. leprae*. Meneses et al. [104] found that urinary MCP-1 was elevated in leprosy patients without any clinical kidney disease, and these levels were much higher in lepromatous polar patients. FoxP3, the main marker of Treg cells, has been found in various forms of leprosy, with and without leprosy reactions. FoxP3+ cells would control acute inflammatory processes, preventing very intense inflammation that could lead to severe nerve losses. FoxP3+ decrease TH1, which may cause bacilli to survive and become distributed in these types of leprosy in patients with MB [110].

## 9. Conclusions

The transmission of *Mycobacterium leprae*, the causative agent of leprosy, is still persistent in society. Various approaches have been used in the past with varying degrees of success, and therefore, the identification of new biomarkers for leprosy is the need of the hour. Numerous studies aimed at the identification of protein(s) as prognostic/diagnostic biomarkers employing proteomics exist. Proteomic profiling helps unravel the connections between various cellular pathways and thus complements both the genomics and traditional biochemical approaches. Proteomics is expected to be the tool of choice for diagnosing patients and searching for therapeutic biomarkers in the years to come.

## Figures and Tables

**Table 1:** List of potential biomarkers identified for the diagnosis of *Mycobacterium leprae* infection (in chronological order).

S. No.	Biomarker	Total No. of Subjects	Sample Type	Applied Technique	Sensitivity/Specificity	Remark	Specimen Collection Location	Year	Ref.
1.	Anti PGL-1 Ab	Hyperimmune anti- *M. leprae* rabbit antiserum, leprosy (TT, LL) patients sera	Serum	ELISA	**Sensitivity**: For Anti PGL-1-IgM Abs LL-96%, TT-62%	Sera were analyzed against both the IgM- and IgG-conjugated reagents, high anti-PGL I IgM was present in LL than TT cases. This assay of IgM against *M. leprae* glycolipid especially in LL cases may result in earlier diagnosis and treatment.	U.S. (Denver-Colorado, Dale, California)	1983	[97]
		Leprosy (114 MB, 85 PB) patients, 42 HHC, 20 EC, 106 ODD, 234 HI **The Netherlands**: 99 HI, 59 other diseases	Whole blood and serum	ML flow test, ELISA	**Sensitivity**: For MB-97.4%, untreated PB-40%, household contacts-28.6%	It is a simple, stable, and rapid tool to categorize the leprosy patients (MB/PB) and identification of leprosy contact patients. It detects IgM antibodies to PGL-1 of *M. leprae.*	Brazil (Manaus), Indonesia (South Sulawesi), Philippines (Cebu), Ghana and Netherlands	2003	[35]
					**Specificity**: For control group-90.2%				
2.	IgG against ESAT-6 (ML0049)	48 Leprosy (PB, MB) patients, 13 untreated TB patients, 14 ODD. patients, 21 HI	Serum	ELISA	**Sensitivity**: For smear positive-82·4%, smear negative-19·4%, both together-41.7%	Results of ESAT-6 based assay was equivalent to anti-PGL-1 antibody detecting ELISA. ESAT-6 act immunologically in leprosy patients and aid in early diagnosis of leprosy, especially in MB cases.	India (Agra, Uttar Pradesh)	2007	[98]
					**Specificity**: 100%				
3.	IFN-γ	*M. leprae* Ags, HI	Whole blood and PBMC supernatant	ELISA PBMC, UCP-LF IFN-γ (ULIGA)	n/a	Analytical sensitivity of ULIGA assay was near about 2 pg/mL IFN-γ in IMDM-HS, thereby 10 folds more sensitive than IFN-γ ELISA. It uses LF-based avidin–biotin capture and detects IFN-γ concentration above 100 pg/mL.	Netherlands (Leiden)	2009	[99]
				Immuno-sandwich assay					
4.	**For T1R**: CXCL10 & IL6	Leprosy (10 T1R, 10 T2R), 29 leprosy patients without reaction	Plasma	The multiplex bead-based technique (Cytokine array)	n/a	These markers aid in differentiating these groups, and provide adequacy in clinical diagnosis and treatment of disease.	Central Brazil (Goiania)	2009	[94]
	**For T2R**: IL7, PDGF-BB & IL6,								
5.	***M. leprae* derived Ags**: Serine-rich 45 kDa protein (45 kDa), ESAT-6, CFP-10, PGL-1	Leprosy (PB, MB) patients	Serum	ELISA	**Sensitivity**: For PB patients, 73%, (providing 36% improvement over conventional PGL-1 based ELISA)	These Ags focused on the detection of PB cases. Antibodies formation against secretory protein ESAT-6 and CFP-10 aid in the detection of early infections and for the monitoring of treatment efficiency.	India	2011	[100]
6.	Abs against PGL-1, LAM and six recombinant *M. leprae* proteins (ML1877, ML0841, ML2028, ML2038, ML0380, ML0050)	Leprosy patients (37 LL, 13 BL, 20 TT/BT, 42 HHC, 23 HI, 30 TB patients	Serum	Western blot, ELISA, ML/lateral flow test	**Sensitivity:** **By lateral flow test for**: -BL/LL-97.4%, TT/BT-40% **By ELISA** a) against ML2028 for: -BL/LL-90%, TT/BT-65% b) against LAM for: -BL/LL-100%, TT/BT-90%, TB-87% c) against ND-O-BSA for: -BL/LL-96%, TT/BT-80%	By Western blot analysis, four of the recombinant proteins, ML1877, ML0841, ML2028, and ML2038, were recognized by sera from all BL/LL and TT/BT patients, while ML2028 and ML2038 showed good response for both MB and PB groups. ML test flow is an important tool to diagnose borderline leprosy. These simple and inexpensive serological test uses the combination of protein Ags in early diagnosis and treatment of disease with high accuracy.	Philippines (Cebu), U.S. (Fort Collins, Colorado)	2011	[101]
					**Specificity:** **By lateral flow test for**: -BT/LL-90.2% **By ELISA** (a) against ML2028—89% (b) against LAM-21% (c) against ND-O-BSA-93%				
7.	MCP-1 (CCL2), MIP-1β (CCL4), IL-1β and IFN-γ induced protein 10 (CXCL10, IP-10)	**Bangladesh**: Leprosy (10 TT/BT) patients, 10 HHC, 10 HI	Whole blood and armadillo-derived *M. leprae* whole cells	ELISA, PBMC	n/a	*M. leprae* recombinant protein induced chemokines/cytokines in leprosy patients and EC. ML2478 and ML0840 induced high IFN-γ concentrations in EC. ML2478 induced higher concentrations of MCP-1, MIP-1b, and IL-1b in patients compared with EC is an important Ag that differentiate between pathogenic and non-pathogenic cases.	Bangladesh (Dhaka), Brazil (Fiocruz Fortaleza), Ethiopia (Addis Ababa), South Korea (Seoul)	2012	[92]
		**Brazil**: Leprosy (10 TT/BT) patients 10 HHC, 10 EC, 10 HI							
		**Ethiopia**: 35 HC, 18 EC (high); 17 EC (low)							
		**Korea**: 10 pulmonary TB, 10 HI							
8.	Abs against LID-1, LAM, ML2028 (Ag85B), ND-O-BSA	**Philippines**: Leprosy {21 MB (2 BL), 10 LL} Patients, 51 HHC	Serum	Western blot, ELISA	n/a	By Western blot analysis, out of all recombinant protein ML2028 and LID-1 Ag showed extreme response in the BL/LL group while weaker response toward other protein Ag. A very strong response was observed to LAM in BT/LL group. The ELISA result showed gradual decay and upraised ND-O-BSA Ag level in high bacillary load patients.	Philippines (Cebu), U.S. (Fort Collins, Colorado)	2012	[64]
9.	IP-10, IL-10, anti-PGL-1 antibodies	**For kinetics of IP-10**: Ethopia (5 BL, 2BT), Netherlands (3 BT), 8 EC	Serum	**Dry-format** **UCP-LFAs for**: IP-10 and anti-PGL-1 antibodies	n/a	The remarkable difference was observed in the ratio of IP-10/IL-10 in sera of all three groups. Results of dry format UCP dry-format UCP-LFAs were equally sensitive as ELISAs.	Ethiopia (Addis Ababa), Netherlands (Leiden)	2014	[102]
		**For cytokine profile**: Ethopia (2 BT, 9 BL, 12 EC)		**Multiplex UCP-LFA format for**: anti-PGL-1 antibodies and IP-10 **ELISA**					
		**Correlation b/w ELISA and UCP-LFAs**: Ethiopia (2 BT, 8 BL, 12 EC)							
10.	CCL18, CCL17, IL-10, CD14	85 Leprosy (38 BT/TT, 3 BB, 44 BL/LL) patients, 6 EC	Serum and skin biopsies	**RT PCR assay for**: Measuring mRNA level in skin lesion **ELISA**	n/a	An elevated level of CCL18 and IL-10 was found in lepromatous while CCL17 and CD14 were found in tuberculoid patient lesions. However, CCL17 and CCL18 were more strongly linked with leprosy polarity as compared to TH1 and TH2 cytokines.	Nepal (Kathmandu)	2014	[103]
11.	MCP-1, MDA	44 Leprosy (14 TT/BT, 19 BB, 11 LL/BL) patients, 15 HI	Urine	**Thiobarbituric acid (TBARS) test for**: MDA.	n/a	Increased levels of MCP-1 and MDA were observed in leprosy patients with no clinical kidney disease. The level of MCP-1 increased in MB patients than PB. MCP-1 and oxidative stress markers indicate high chances of developing kidney disease in leprosy patients.	Brazil (Fortaleza)	2014	[104]
				**ELISA for**: MCP-1					
12.	IFN-γ, IP-10-, IL-17- VEGF, IL-10	**Bangladesh**: Leprosy patient (31 BL/LL, 20 RR) Patient, 20 EC	Whole blood and serum	ELISA, PBMC	n/a	PBMC peaked stimulation occurs by IFN-γ-, IP-10-, IL-17, and VEGF through *M. leprae* Ag that diagnosed T1R. However, a decline in IL-10 level was observed in T1R while it was elevated after treatment. The ratio of these biomolecules (pro-inflammatory cytokines with IL-10) allows early diagnosis of T1R and its cure.	Bangladesh (Dhaka), Brazil (Uberlandia), Ethiopia and Nepal (Kathmandu)	2015	[105]
		**Brazil**: Leprosy patient (23 BL/LL, 25 RR) Patient, 20 EC							
		**Ethiopia**: Leprosy patient (11 BL/LL, 25 RR) Patient, 15 EC							
		**Nepal**: Leprosy patient (20 BL/LL, 13 RR) Patient, 20 EC							
13.	Abs against PGL-1 LID-1	**Cohort 1 (Philippine)**: 127 LL/BL, 24 BT/TT, 4 LL	Serum	**ELISA, Ab Rapid test (Gold-LFA) for**: detection of IgG antibodies directed against LID-1	**Philippine (MB with low BI) Sensitivity**: 94% UCP-LFA, 78% gold LFA **Specificity**: 100% by both	Comparison of two field-friendly assays i.e., Gold-LFA and UCP-LFA aid in the detection of *M. leprae*-specific humoral immune responses. The accuracy of UCP-LFA assay in MB patients (BI+) was more than Gold-LFA. PGL-1 and LID-1 both are reported in MB patients. In the Bangladesh cohort, most of the PB patients were found negative by using both these methods along with ELISA against PGL-1.	Philippine (Cebu), Bangladesh (Nilphamari), Brazil (Pará)	2017	[106]
					**Bangladesh (MB with high BI) Sensitivity**: 41% UCP-LFA, 44% gold LFA				
		**Cohort 2 (Bangladesh)**: 34 MB (8 BL/LL, 26 BT), 45 PB (41 BT, 4 TT), 54 HHC, 50 HHC & BCG		**PGL-1 UCP-LFA for** detection of IgM antibodies directed against PGL-1.					
		**Cohort 3 (Brazil)**: 60 hyperendemic area							
					**Brazil Sensitivity**: 28% by both				
14.	C1q (C1qA, C1qB, and C1qC)	30 untreated ENL, 30 non-reactional LL	Whole blood and skin biopsies	qPCR, ELISA	n/a	C1q was used as a potential diagnostic marker for active ENL reactions, and it was also used for monitoring ENL treatment. qPCR determines the three components of C1q mRNA expression in blood and dermal biopsies.	Ethiopia (Addis Ababa)	2018	[107]
15.	anti-PGL-1 IgM antibody, IP-10, CCL4, CRP	**Cohort 1 (Brazil)**: Leprosy (30 LL/BL, 41 BT/TT) patients, 103 HHC, 237 EC	Whole blood	UCP-LFA	**Sensitivity**:	This technique ease in rapid testing based on selected biomarkers using finger stick blood (FSB). For LL/BL and BT/TT leprosy patients, IP-10 was the most significant marker for identification. For LL/BL cases, anti-PGL-1 IgM and CRP are prominent for diagnosis and CCL4 is prominent for the detection of BT/TT patients.	Brazil (Pará), China (Qianxinan and the Guiyang prefecture), Ethiopia (Kokosa Woreda)	2018	[108]
					**for LL/BL patients**: 91% (China), 97% (Brazil), 75% (Ethiopia)				
		**Cohort 2 (China)**: Leprosy (47 LL/BL, 4 BT/TT) patients, 87 HHC, 56 EC							
					**for BT/TT patients**: 80% (China), 71% (Brazil), 75% (Ethiopia)				
		**Cohort 3 (Ethiopia)**: Leprosy (17 LL/BL, 4 BT/TT) patients, 24 HHC, 25 EC							
16.	ApoA1 (Apolipoprotein A1), IL-1Ra, S100A12 (calgranulin C)	**Cohort 1**: Leprosy (34 MB, 45 PB) patients, 54 HHC, 51 EC	Whole blood and plasma	Multiplex bead arrays, ELISAs and UCP-LFAs	**Sensitivity**: UCP-LFAs 86%	Along with these three new biomarkers, five (CCL4, CRP, IL-10, IP-10, αPGL-1 IgM) previously identified biomarkers were also confirmed. Overnight WBAs stimulation increased specificity for IL-10, IL-1Ra and CCL4 markers. The rest of the other markers can be detected in plasma for rapid POC tests, LFAs utilized these markers in the detection of MB and PB patients.	Bangladesh (Nilphamari, Rangpur, Panchagar, and Thakurgaon)	2019	[71]
		**Cohort 2**: Leprosy (27 MB, 28 PB), patients, 27 EC			**Specificity**: UCP-LFAs 90%				
		**Cohort 3**: Leprosy (21 MB, 15 PB) patients, 28 EC							
17.	CCL4, CRP, IL-10, IP-10, αPGL-1 IgM	**Bangladesh**: Leprosy (27 MB, 15 PB) patients, 27 HHC, 12 EC	Fingerstick blood (FSB) and serum	UCP-LFAs	n/a	Minimally invasive and user-friendly quantitative UCP-LF along with FSB aid in the detection of the biomarker for *M. leprae* infection. All MB cases were perfectly identified by αPGL-1 FSB test conferring a good quantitative correlation with the BI.	Bangladesh (Nilphamari), Brazil (Marituba), South Africa (Cape Town), and the Netherlands (Rotterdam)	2019	[109]
		**Brazil**: Leprosy (8 MB, 4 PB) patients, 4 HHC, 5 ODD							
		**South Africa**: 4 MB, 1 HI							
		**The Netherlands**: 3 MB, 6 PB, 1 ODD							
18.	FoxP3	Leprosy (PB, MB, T1R, T2R) patients, EC (10 individuals selected for each case)	Whole blood and plasma	ELISA, PBMC	n/a	ELISA is an inexpensive method involved in the detection of the FoxP3 marker. A rise in FoxP3+ cells in T1R patients could be advantageous to the host as a protection mechanism, while the decline in Th1 immune response by FoxP3+ cells in MB patients leads to survival and dispersion of the bacilli.	Brazil (Goiânia)	2019	[110]

Abbreviations: TT: tuberculoid leprosy; BT: borderline tuberculoid; BB: mid-borderline; BL: borderline lepromatous; LL: lepromatous leprosy; PB: paucibacillary; MB: multibacillary; ENL: Erythema nodosum leprosum; T1R, T2R: leprosy type 1 and 2 reaction; RR: reversal reaction; EC: endemic controls; HHC: household contacts; HI: healthy individuals; ODD: other dermatological diseases; Abs: antibodies Ags: antigens; BI:bacterial indices; CRP: C-reactive protein; CCL: chemokine ligand; CXCL10: CXC-chemokine 10; ELISA: enzyme linked immunosorbent assay; ESAT-6: early secreted antigenic target-6; IL-1β IL6, IL7: interleukin; IP- 10: interferon gamma- induced protein; IFN-γ: interferon gamma; IMDM-HS: Iscove’s modified dulbecco medium human serum; LAM: lipoarabinomannan; LID-1: leprosy IDRI diagnostic; MCP1: monocyte chemoattractant protein-1; MDA: malondialdehyde; MIP-1β: macrophage inflammatory protein-1β; ML flow: lateral flow test; ND-O-BSA: natural disaccharide octyl bovine serum albumin; PBMC: peripheral blood mononuclear cell; PDGF-BB: platelet-derived growth factor BB; PGL-1: phenolic glycolipid 1; TB: tuberculosis; UCP- LFA: upconverting phosphor-lateral flow assays; UCP-LF IFN-γ: up-converting phosphor-lateral flow; VEGF: vascular endothelial growth factor; WBAs: whole blood assay.
